# Supplementary material for: Predictive Model for Preeclampsia Combining sFlt-1, PlGF, NT-proBNP, and Uric Acid as Biomarkers
Source: J Clin Med. 2023 Jan 5;12(2):431. doi: 10.3390/jcm12020431 (PMC9866466; doi:10.3390/jcm12020431)
Supplement: Supplementary file 1 [file jcm-12-00431-s001.zip › jcm-2098523-supplementary.pdf]

SUPPLEMENTARY MATERIALS

SUPPLEMENTARY METHODS

Predictive models

A random forest-based supervised machine-learning model was used to predict the risk level of PE (low, moderately low, moderately high, or high risk) of a patient with clinical suspicion. We have set that the patient will be negative if none of the patient's repeated measurements have a moderate-high risk at least. Otherwise, the patient is considered positive. True positive and true negative patients are those coincident with the final decision of the clinician.

The prediction of a random forest model is built by averaging the predictions of many randomized decision trees. A decision tree is a model that is able to derive a medical guideline for the assessment or management of a patient from the experience of some clinicians that is collected in a database. However, the model could either easily end up developing an accurate guideline though inconsistent on average, or a consistent one though inaccurate on average. A random forest is an ensemble model that involves bagging (bootstrap aggregating); it selects many bootstrap samples from the database and fits a decision tree on each, in order to make a guideline as accurate as consistent on average.

The module sklearn.ensemble.RandomForestClassifier of the library sklearn, which includes several free software machine-learning models for the Python programming language, was used.

Table S1. Decision thresholds.

**Early-preterm PE**	sFlt-1/PlGF ratio	sFlt-1/PlGF + proBNP + urid acid
**Rule out**		
Low	0-16.0	0-15.5
Moderately low	16.0-38.4	15.5-41.5
**Rule in**		
Moderately high	38.4-59.3	41.5-65.4
High	59.3-100	65.4-100

**Late-preterm PE**	sFlt-1/PlGF ratio	sFlt-1/PlGF + proBNP + urid acid
**Rule out**		
Low	0-14.4	0-13.2
Moderately low	14.4-21.6	13.2-33.2
**Rule in**		
Moderately high	21.6-31.0	33.2-54.2
High	31.0-100	54.2-100

SUPPLEMENTARY FIGURES

A)

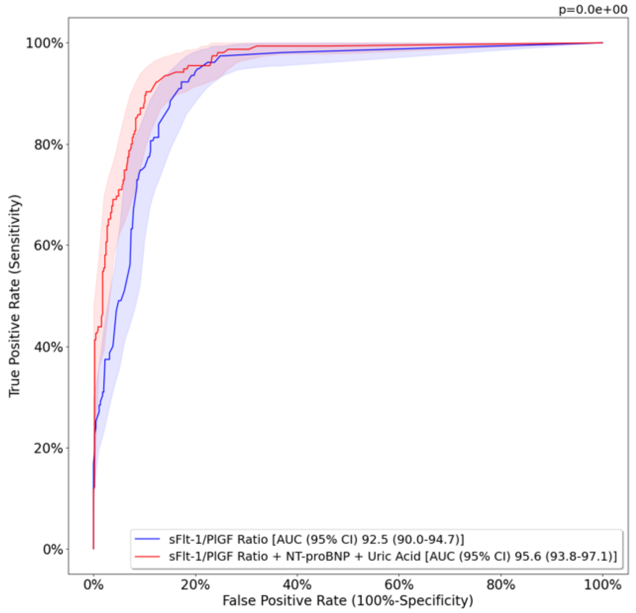

B)

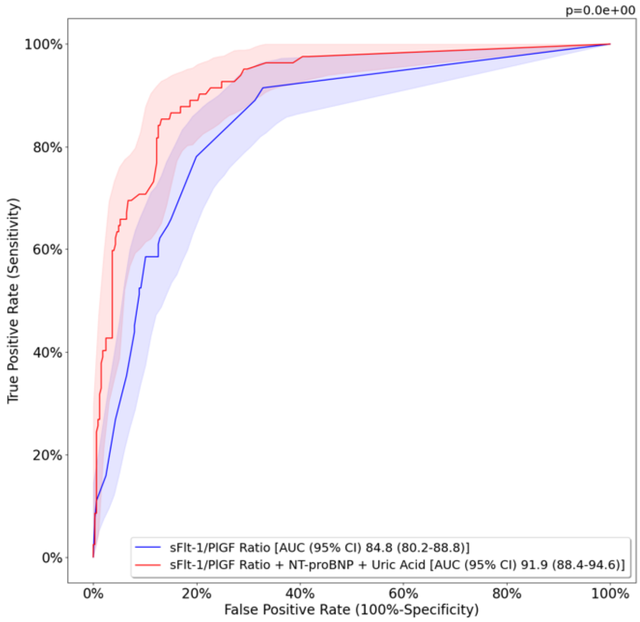

sFlt-1, soluble fms-like tyrosine kinase 1; PlGF, placental growth factor; NT-proBNP, N-terminal pro-brain natriuretic peptide; AUC, area under the curve; CI, confidence interval.

Preterm PE was defined as PE below 37^{+0} weeks.

Figure S1. Performance of studied models for predicting preterm PE. (**A**) Prediction of early-preterm PE and (**B**) prediction of late-preterm PE.

A)

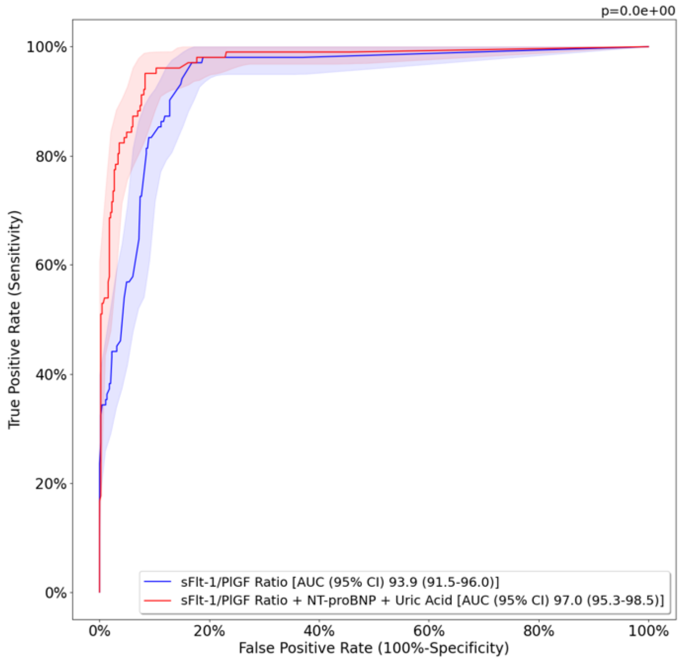

B)

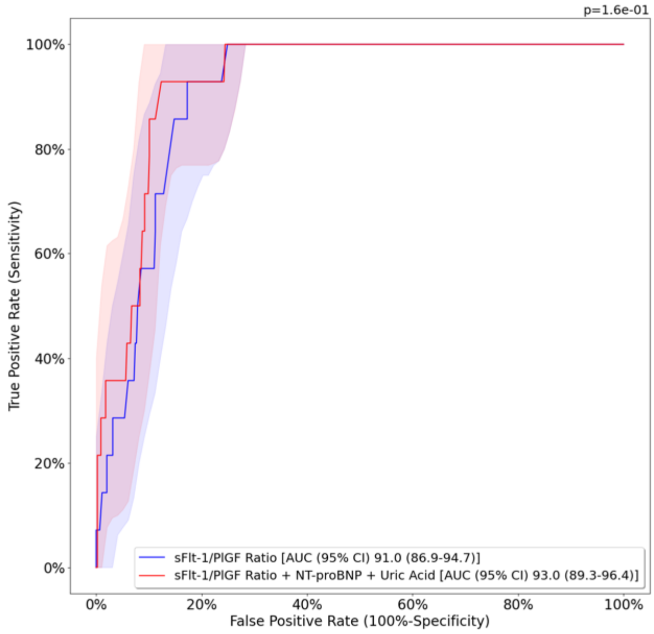

sFlt-1, soluble fms-like tyrosine kinase 1; PlGF, placental growth factor; NT-proBNP, N-terminal pro-brain natriuretic peptide; AUC, area under the curve; CI, confidence interval.

Early-preterm PE was defined as PE below 34^{+0} weeks.

Figure S2. Performance of studied models for predicting early-preterm PE within 1 and 3 weeks to delivery. (A) Prediction of early-preterm PE within 1 week to delivery and (B) prediction of early-preterm PE within 3 weeks to delivery.

A)

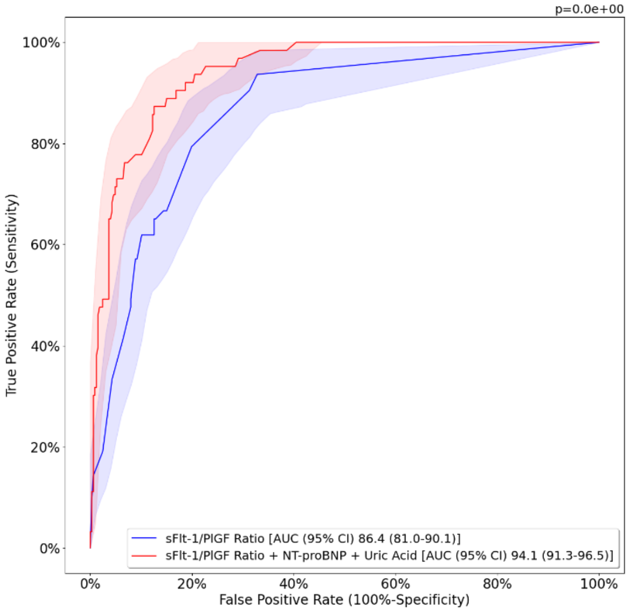

B)

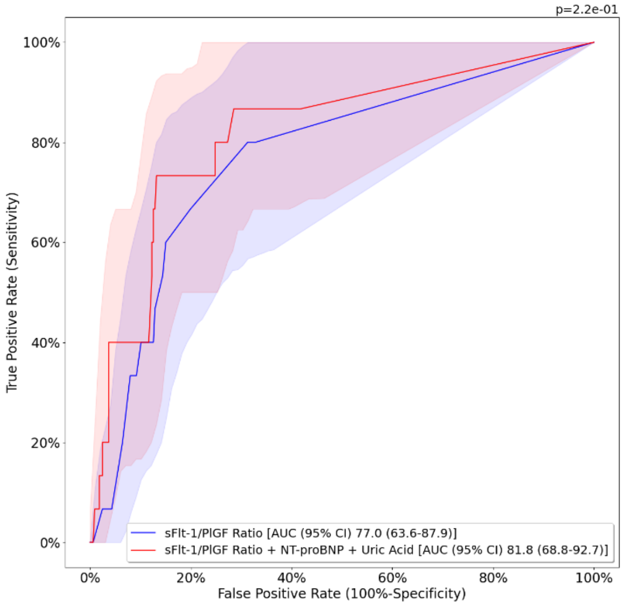

sFlt-1, soluble fms-like tyrosine kinase 1; PlGF, placental growth factor; NT-proBNP, N-terminal pro-brain natriuretic peptide; AUC, area under the curve; CI, confidence interval.

Late-preterm PE was defined as PE between 34^{+0} and 36^{+6}weeks.

Figure S3. Performance of studied models for predicting late-preterm PE within 1 and 2 weeks to delivery. (A) Prediction of late-preterm PE within 1 week to delivery and (B) prediction of late-preterm PE within 2 weeks to delivery.
